# The long-term curative effect analysis of trans-articular plate combined with Kirschner wires in the treatment of fracture-dislocation of the fifth carpometacarpal joint

**DOI:** 10.3389/fsurg.2022.1088483

**Published:** 2023-01-10

**Authors:** Ying Zhou, Ye Wu, Yinjun Huang, Renguo Xie, Song Gu

**Affiliations:** ^1^ Department of Radiology, Shanghai General Hospital, Shanghai Jiao Tong University School of Medicine, Shanghai, China; ^2^ Department of Nursing, Shanghai General Hospital, Shanghai Jiao Tong University School of Medicine, Shanghai, China; ^3^ Trauma Center, Shanghai General Hospital, Shanghai Jiao Tong University School of Medicine, Shanghai, China

**Keywords:** carpometacarpal joint, internal fixation, fracture-dislocation, kirschner wire, long-term

## Abstract

**Purpose:**

To evaluate the long-term curative effect analysis of trans-articular plate combined with Kirschner wires in the treatment of fracture-dislocation of the fifth carpometacarpal joint.

**Methods:**

From July 2016 to September 2021, 21 patients with fracture-dislocation of the fifth carpometacarpal joint were treated with trans-articular plate combined with Kirschner wires internal fixation. Each patient's gender, age, dominant hand, injured hand, trauma mechanism, the time between injury and surgery, the range of motion of the bilateral wrist in flexion, extension, radial deviation and ulnar deviation, grip strength of each side, the time of return to work, and follow-up time were recorded. The QDASH score and Cooney wrist function score were used to evaluate the postoperative function. The VAS system was used to evaluate postoperative pain.

**Results:**

The follow-up time was 37.0 ± 19.0 months and the time between injury and surgery was 1.3 ± 0.5 days. In the injured side and the contralateral side, the range of motion of the wrist flexion were 58.3 ± 4.0° and 60.5 ± 3.1°, the range of motion of the wrist radial deviation were 25.7 ± 3.3° and 26.9 ± 2.9°, the range of motion of the wrist ulnar deviation were 28.1 ± 3.7° and 29.5 ± 3.1° respectively with no significant difference. The range of motion of the wrist extension (54.0 ± 3.4°) in the injured side was smaller than that in the contralateral side (56.7 ± 3.7°) with significant difference. The grip strength of the injured side and the contralateral side were 96.1 ± 9.5 LB and 100.7 ± 9.7LB respectively with no significant difference. The QDASH score was 3.8 ± 1.8, Cooney wrist function score was 94.5 ± 4.2, VAS score was 1.0 ± 0.8 and the time of return to work was 5.1 ± 0.9 weeks. In the 21 cases, no postoperative complications such as incision infection, failure of internal fixation, fracture nonunion or fracture malunion occurred.

**Conclusion:**

The method of trans-articular plate combined with Kirschner wires is one of the alternative treatments for the fracture-dislocation of the fifth carpometacarpal joint. The long-term follow-up results were satisfactory.

## Introduction

Fracture-dislocation of the fifth metacarpal base is infrequent in hand injuries (1–3). Once the ulnar part of hand suffers injury, the fifth metacarpal base fracture and the fifth carpometacarpal joint dislocation may occur at the same time. However, this kind of injury is very subtle, easy to miss and difficult to treat (4, 5). If the proper treatment is not been taken, it is prone to result in fracture malunion, persistent pain, weakened grip strength, carpometacarpal joint arthritis and other complications. The result of conservative treatment is not satisfactory and surgery has become the mainstay of treatment (6, 7). How to treat the fracture-dislocation of the fifth carpometacarpal joint has always been a hot topic in the field of hand surgery. At present, the commonly used surgical methods include Kirschner wire internal fixation (8), open reduction plate internal fixation (9, 10) and external fixation (11), but all of them have shortcomings. The poor strength of Kirschner wire internal fixation can not guarantee the safety of early function exercise; the fracture of the metacarpal base is close to the carpometacarpal joint, which results in few space for proximal screws of plate internal fixation; external fixation will affect the daily life of patients after surgeries because the exposed nails might be scratched and cause pain.

In view of this kind of injury, we proposed a trans-articular plate combined with Kirschner wires method to treat it a few years ago and achieved good results in preliminary studies (12). However, due to the shortcomings of the previous study such as small sample size and short follow-up time, this approach was still not convincing. To explore the long-term efficacy of this method we conducted further in-depth research. The aim of this study was to retrospectively analyze the long-term curative effect of trans-articular plate combined with Kirschner wires in the treatment of fracture-dislocation of the fifth carpometacarpal joint.

## Materials and methods

From July 2016 to September 2021, we treated 21 patients with fracture-dislocation of the fifth carpometacarpal joint, all of whom were treated with trans-articular plate combined with Kirschner wires internal fixation. The inclusion criteria included that (1) closed and fresh fifth metacarpal base fracture; (2) the fracture line involved the carpometacarpal joint surface; (3) the fifth carpometacarpal joint was dislocated; (4) there was no other injury in the hand. The exclusion criteria included that (1) open fifth metacarpal base fracture; (2) old fifth metacarpal base fracture; (3) there was no dislocation in the fifth carpometacarpal joint; (4) there was other injury in the hand. Table 1 lists the demographic data of the 21 patients.

**Table 1:** Demographic data of patients (*n* = 21).

Parameter	Value
**Gender (*n*, %)**	
Male	19, 90.5
Female	2, 9.5
**Age (years)**	36.0 ± 8.6
**Dominant hand (*n*)**	
Right	17
Left	4
**Injured hand (*n*)**	
Right	18
left	3
**Trauma mechanism (*n*)**	
Traffic accident	7
Sports injury	9
Fall down	5
**Time between injury and surgery (days)**	1.3 ± 0.5

### Surgical technique

Local or general anesthesia was adopted and a balloon tourniquet was placed in the middle of the upper arm to reduce bleeding. A 5 cm or so incision was made in the dorsal ulnar side of the hand. The nerves and tendons needed to be protected carefully, and then the carpometacarpal joint capsule was opened. After the little finger was pulled distally, the articular surface was completely exposed. The soft tissues trapped in the joint were cleared and the attachment points of the tendons and ligaments were preserved. After completely exposing the fracture-dislocation of the fifth carpometacarpal joint, one or two Kirschner wires with diameter of 0.8 or 1.0 mm were selected to fix the fracture fragments to restore the articular surface and achieve the anatomical reduction as far as possible. C-arm fluoroscopy was used to confirm that the reduction was satisfactory. A L-shaped plate (Zimmer Biomet, Warsaw, Indiana, United States) was fixed across the carpometacarpal joint, with proximal screws in the hamate bone and distal screws in the fifth metacarpal. C-arm fluoroscopy was performed again to check the fracture reduction and satisfactory position of the plate and screws. The incision was sutured with 4-0 Coated Vicryl Plus Antibacterial Suture (Ethicon, Somerville, NJ, United States) and wrapped with appropriate pressure.

### Postoperative management

2 weeks after the operation, the suture was removed and the function exercise started. All patients underwent function exercise under the guidance of the same group of physiotherapists. The function exercises of the interphalangeal and metacarpophalangeal joint were started after the suture was removed, and the wrist joint function exercises were started 4 weeks after the operation. X-rays were examined every 4–5 weeks after the operation. Once the fracture got healed, the removal of the plate and Kirschner wires was performed under local anesthesia and the function exercise of full range motion of each joint was continued.

### Outcome evaluation

Each patient's gender, age, dominant hand, injured hand, trauma mechanism, the time between injury and surgery, the range of motion of the bilateral wrists in flexion, extension, radial deviation and ulnar deviation, grip strength of each side, the time of return to work, and follow-up time were recorded. Postoperative complications such as the failure of internal fixation, fracture nonunion and fracture malunion were recorded. Grip strength was measured with a Jamar dynamometer (Baseline Hydraulic Hand Dynamometers; Fabrication Enterprises, White Plains, NY, United States). According to the QDASH (the Quick Disability of the Arm, Shoulder and Hand) score and Cooney wrist function score, we evaluated the postoperative function. The VAS (Visual Analogue Scale) system was used to evaluate postoperative pain. Another group of surgeons who did not participate in the surgeries performed these assessments. The mean and standard deviation of the time between injury and surgery, follow-up time, the time of return to work, the range of motion and grip strength were calculated. Meanwhile, the range of motion and the grip strength of both sides were calculated, and one-way analysis of variance was carried out. *P* value of <0.05 was considered statistically significant, and SPSS 23.0 software was used for statistical analysis.

## Results

**Figure 1:**
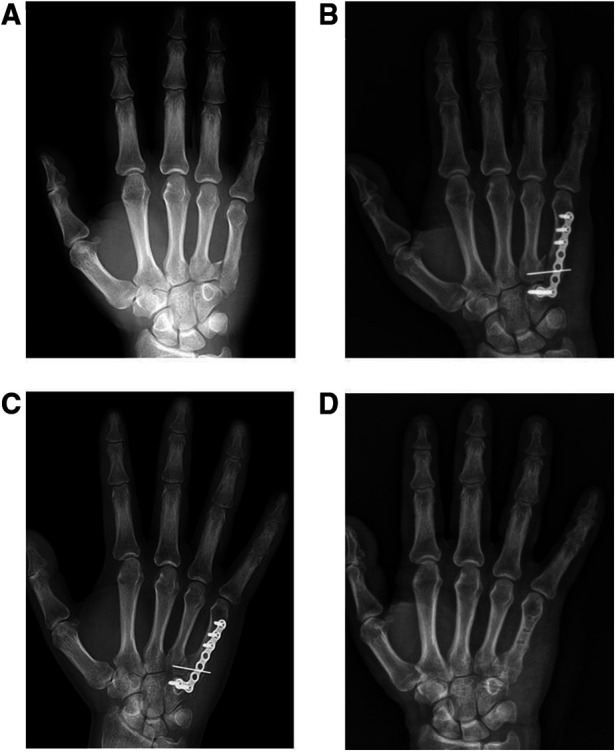
The treatment process of a patient with fracture-dislocation of the fifth carpometacarpal joint of the right hand. (**A**) The preoperative anteroposterior x-ray. (**B**) The postoperative anteroposterior x-ray the day after internal fixation surgery. (**C**) The postoperative anteroposterior x-ray 10 weeks after internal fixation surgery. (**D**) The postoperative anteroposterior x-ray the day after internal fixation removal surgery.

**Figure 2:**
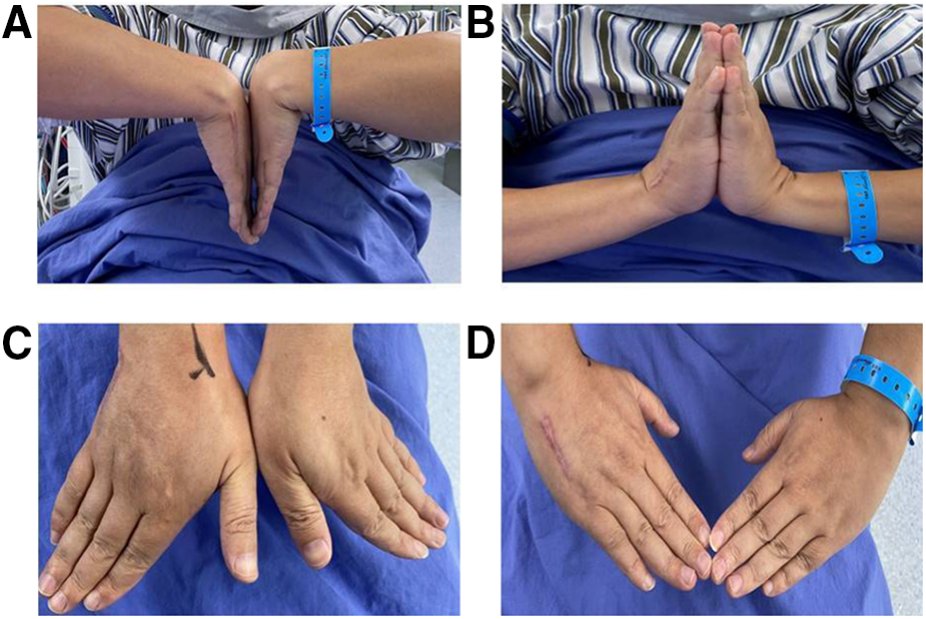
The range of motion of bilateral wrists of a patient in the last follow-up. (**A**) The appearance of the extension position. (**B**) The appearance of the flexion position. (**C**) The appearance of ulnar deviation position. (**D**) The appearance of radial deviation position.

The follow-up time was 37.0 ± 19.0 months (ranging from 9 to 65 months) and the time between injury and surgery was 1.3 ± 0.5 days (ranging from 1 to 2 days). In the injured side and the contralateral side, the range of motion of the wrist flexion were 58.3 ± 4.0° and 60.5 ± 3.1°, the range of motion of the wrist radial deviation were 25.7 ± 3.3° and 26.9 ± 2.9°, the range of motion of the wrist ulnar deviation were 28.1 ± 3.7° and 29.5 ± 3.1° respectively, and the differences were not statistically significant. The range of motion of the wrist extension (54.0 ± 3.4°) in the injured side was smaller than that in the contralateral side (56.7 ± 3.7°) with significant difference. The grip strength of the injured side and the contralateral side were 96.1 ± 9.5 LB and 100.7 ± 9.7 LB respectively and the differences were not statistically significant (Table 2). The QDASH score was 3.8 ± 1.8, Cooney wrist function score was 94.5 ± 4.2, VAS score was 1.0 ± 0.8 and the time of return to work was 5.1 ± 0.9 weeks (Table 3). In the 21 cases, no postoperative complications such as incision infection, failure of internal fixation, fracture nonunion or fracture malunion occurred. A typical case was shown in Figures 1, 2.

**Table 2:** Grip strength and range of motion of patients (*n* = 21).

	TIS	TCS	*P* value
**Grip strength (LB)**	96.1 ± 9.5	100.7 ± 9.7	0.136
**Range of motion (°)**			
Flexion	58.3 ± 4.0	60.5 ± 3.1	0.059
Extension	54.0 ± 3.4	56.7 ± 3.7	0.021
Radial deviation	25.7 ± 3.3	26.9 ± 2.9	0.223
Ulnar deviation	28.1 ± 3.7	29.5 ± 3.1	0.184

TIS, the injured side; TCS, the contralateral side.

**Table 3:** Function outcome of patients (*n* = 21).

Parameter	Value
Follow up time (months)	37.0 ± 19.0
QDASH score	3.8 ± 1.8
Cooney wrist function score	94.5 ± 4.2
VAS score	1.0 ± 0.8
Return to work (weeks)	5.1 ± 0.9

## Discussion

Fracture-dislocation of the fifth carpometacarpal joint is commonly missed (13–15), so that the true incidence is difficult to record. Treatment options for this injury mainly include non-operative management, closed reduction with Kirschner wire fixation and open reduction with internal fixation. In recent years, some scholars have also reported some improved methods and achieved good results (16, 17). Up to now, Which treatment strategy is more effective has been controversial (18–21).

Our research group had proposed a new method of trans-articular plate combined with Kirschner wires for the treatment of fracture-dislocation of the fifth metacarpal base several years ago, and had achieved preliminary good results. This method can restore the articular surface by Kirschner wires fixation and avoid the risk of malunion and arthritis as much as possible. Plate and screws internal fixation can enhance the fixation strength and meet the need of early function exercise. At the same time, all internal fixators are located in the hand and will not affect the daily life of patient after operation. However, the previous study only enrolled 10 patients and the follow-up time was short. To enhance the persuasion, we continued to carry out this research to further explore the long-term efficacy of this strategy.

In the present study, we found there was no significant difference in grip strength between the injured and contralateral wrists of the patients. In terms of range of motion, there was no significant difference in flexion, radial deviation and ulnar deviation between the two sides. The range of wrist extension of the affected side was slightly smaller than that of the healthy side. The reason for this might be related to the lack of function exercise and tendon adhesion after operation. The decreased bone density in postoperative x-ray also proved that there might be a certain degree of disuse osteoporosis. The fifth carpometacarpal joint is a joint with micromotion because of its anatomical features (22). Although the trans-articular plate fixation did temporarily resulted in some loss of range of motion, the trans-articular plate also provided a reliable fixation strength for early function exercise at the same time, no internal fixation failure or fracture malunion occurred in all patients. All patients started function exercises with full range of motion of every interphalangeal and metacarpophalangeal joint after the fracture healed and the internal fixation was removed to avoid the tendon adhesion and joint stiffness as much as possible. The results of this study also showed that the wrist function and the degree of pain of all patients were satisfactory, and all patients returned to work about five weeks after internal fixation operations. The research results with larger sample size and longer follow-up time also showed that the effect of this method is reliable. For the fracture-dislocation of the fifth carpometacarpal joint, it is very important to restore the articular surface and provide reliable fixation strength. The quality of articular surface recovery is closely related to fracture malunion, arthritis and pain, and enough fixation strength can ensure the safety of early function exercise which is related to satisfactory grip strength and range of motion. In our treatment strategy, we combine the advantages of Kirschner wires, which can restore articular surface, and plate, which provides enough fixation strength.

The advantages of trans-articular plate combined with Kirschner wires in the treatment of fracture-dislocation of the fifth carpometacarpal joint are as following: (1) the fracture fragments of the fifth metacarpal base are too small to provide enough space for the proximal screws of the plate in the conventional internal fixation method, while trans-articular plate fixation can solve this problem, and the hamate bone can meet the placement requirement of the proximal screws. (2) During the operation, the fracture fragments and articular surface of carpometacarpal joint could be directly exposed after the little finger is pulled to the distal end. The operation under direct view could ensure satisfactory reduction and effectively avoid the injuries of soft tissues such as nerves, tendons and so on. (3) Although the trans-articular fixation temporarily limited the range of motion of the carpometacarpal joint, the satisfactory range of motion of the wrist can be restored with timely removal of the internal fixation and the standardized function exercise. Of course, this method also has some disadvantages, such as the need for a second operation and increased cost of treatment.

During the operation we should pay special attention to these problems: (1) the extensor tendon, ulnar nerve and the attachment point of the ligament at the base of the metacarpal bone should be protected during the operation. (2) After the fracture was exposed, the hematoma trapped in the joint should be thoroughly cleaned, which was beneficial to fracture reduction and dislocation correction. (3) On the premise of ensuring fracture reduction and articular surface recovery, surgeons needed to avoid the Kirschner wires penetrating the articular surface to reduce the damage to the articular surface as much as possible. (4) C-arm fluoroscopy must be used to confirm that the proximal screws of the plate were not located in the joint space.

The lack of comparison with other surgical methods is the main deficiencies of this study. In the following work, the sample size will be further increased and comparative studies will be conducted as well.

## Data Availability

The original contributions presented in the study are included in the article/Supplementary Material, further inquiries can be directed to the corresponding author.
